# Epsilonproteobacteria in Humans, New Zealand

**DOI:** 10.3201/eid1810.120369

**Published:** 2012-10

**Authors:** Susan Bullman, Daniel Corcoran, James O’Leary, Deirdre Byrne, Brigid Lucey, Roy D. Sleator

**Affiliations:** Cork Institute of Technology, Cork, Ireland (S. Bullman, B. Lucey, R.D. Sleator);; and Cork University Hospital, Cork (D. Corcoran, J.O’Leary, D. Byrne, B. Lucey)

**Keywords:** Campylobacter, ureolyticus, Epsilonproteobacteria, Helicobacter, Arcobacter, PCR, epidemiology, volunteers, diarrhea, molecular, diagnostic, bacteria, New Zealand

**To the Editor:** Cornelius et al*.* (*1*) addressed the potential of *Campylobacter ureolyticus* as an emerging pathogen by conducting a molecular study on 128 diarrheal specimens and 49 fecal samples from healthy volunteers**.** Reporting the identification of *C. ureolyticus* in 12 (24.5%) of 49 healthy volunteers, a number that they compared with our finding of 349 (23.8%) from *Campylobacter* spp.–positive samples (*2*), the authors concluded that *C. ureolyticus* species “are unlikely causes of diarrhea,” an assertion with which we take issue.

This interpretation does not take into account that our screening involved 7,194 symptomatic patients: a sample size 40× greater than that of Cornelius et al*.* In this context, the likely carriage rate for *C. ureolyticus* is 1.15%. Also, our assay, which has a limit of detection in the picomolar range, is likely comparable with, if not greater than, that of Cornelius et al. (*1*).

Accounting for variations in geographic location and detection methods, a detection rate of 24.5% in healthy volunteers (overall detection rate 14.7%) is high in contrast to our reported rate of 1.15%. One possible explanation for this discrepancy is that Cornelius et al. “did not specifically exclude volunteers who had had gastrointestinal disturbances in the 10 days before sampling,” Campylobacter can be shed in feces for <4 weeks after infection. Also, Cornelius et al. (*1*) noted the possibility of “genetically distinct but phenotypically indistinguishable genomospecies differing in their pathogenic potential” to account for the presence of the emerging pathogen *C. concisus* in healthy volunteers and patients with diarrheal illness. This may also apply for *C. ureolyticus*.

We reported a strong seasonal prevalence of *C. ureolytcius* and a bimodal age distribution (*2*). The lack of any related details from Cornelius et al. may undermine their reported detection rates. These factors strongly suggest that the statement, “these species are unlikely causes of diarrhea,” should, at the very least, be taken under advisement.
