# Supplementary material for: Epsilonproteobacteria in Humans, New Zealand
Source: Emerg Infect Dis. 2012 Oct;18(10):1709–10. doi: 10.3201/eid1810.120369 (PMC3479867; doi:10.3201/eid1810.120369)
Supplement: Related texts

# Reply: Epsilonproteobacteria in Humans, New Zealand

**DOI:** 10.3201/eid1810.121122

**Authors:** Stephen L.W. On, Angela J. Cornelius

**Affiliations:** Institute of Environmental Science and Research, Christchurch, New Zealand

**In Response:** In response to the letter by Bullman et al. (*1*), a major aspect of our study (*2*) was to compare epsilonproteobacterial populations in healthy persons and those who have diarrhea. We have not examined as many diarrheal samples as Bullman et al. (*3*). However, in contrast with their study, we have examined samples from persons with no evident disease manifestations. Because the presence of an agent during disease is not proof of causation, we believed that a baseline for comparison was needed. *Campylobacter ureolyticus* was found in a greater proportion of samples from healthy persons (24%) than samples from persons who had diarrhea (11%) (p = 0.041, by χ^2^ test).

Samples from healthy persons were tested on 2 occasions: 18 samples in September 2007 (New Zealand spring) at the Institute of Environmental Science and Research, Christchurch, in the workplace, and 31 samples in June 2009 (New Zealand winter), at Christchurch Hospital under the guidance of a clinician. We have no reason to believe any of the workplace samples were provided when volunteers had diarrhea, particularly considering our workplace guidelines and staff characteristics. In each testing round, 6 fecal samples had positive test results for *C. ureolyticus.* These periods equate to the peak and trough periods described by Bullman et al. (*3*). We were unable to provide many details regarding sampling in our paper because of space constraints.

Considering our baseline comparisons of healthy persons with those who had diarrhea, we affirm our conclusions are reasonable and that *C. ureolyticus* is an unlikely cause of acute diarrheal disease. Similarly, *C. ureolyticus* (previously classified as *Bacteroides* *ureolyticus*) has been detected in patients with Crohn’s disease and in controls (*4*). However, different subtypes or undescribed subspecies may be pathogenic: some strains exhibit certain pathogenic characteristics in vitro (*5*) and others yield amplified fragment length polymorphism profiles that are visually quite distinct from others (*6*). Host factors also cannot be discounted.

## References

[1] Bullman S, Corcoran D, O’Leary J, Byrne D, Lucey B, Sleator R. Epsilonproteobacteria in humans, New Zealand [letter]. Emerg Infect Dis [Internet]. 2012 Oct [cited 2012 Aug 17]. 10.3201/eid1810.120369PMC347986723017215

[2] Cornelius AJ, Chambers S, Aitken J, Brandt SM, Horn B, On SLW. Epsilonproteobacteria in humans, New Zealand. Emerg Infect Dis. 2012;18:510–2.10.3201/eid1803.110875PMC330957422377283

[3] Bullman S, Corcoran D, O’Leary J, O’Hare D, Lucey B, Byrne D, Emerging dynamics of human campylobacteriosis in southern Ireland. FEMS Immunol Med Microbiol. 2011;63:248–53.10.1111/j.1574-695X.2011.00847.x22077228

[4] Zhang L, Man SM, Day AS, Leach ST, Lemberg DA, Dutt S, Detection and isolation of *Campylobacter* species other than *C. jejuni* from children with Crohn’s disease. [PubMed]. J Clin Microbiol. 2009;47:453–5.1905218310.1128/JCM.01949-08PMC2643684

[5] Burgos-Portugal JA, Kaakoush NO, Raftery MJ, Mitchell HM. Pathogenic potential of *Campylobacter ureolyticus.* Infect Immun. 2012;80:883–90.10.1128/IAI.06031-11PMC326431722124656

[6] Vandamme P, Debruyne L, De Brandt E, Falsen E. Reclassification of *Bacteroides ureolyticu*s as *Campylobacter ureolyticus* comb. nov., and emended description of the genus *Campylobacter.* Int J Syst Evol Microbiol. 2010;60:2016–22.10.1099/ijs.0.017152-019801389
